# Pretreatment MRI radiomics for predicting pathological Miller-Payne grading in breast cancer following neoadjuvant chemotherapy

**DOI:** 10.1186/s40644-026-00990-5

**Published:** 2026-01-16

**Authors:** Chengliu Bi, Ao Chen, Fengming Ran, Zaoxiu Hu, Shaomei Sun, Ruolan Wang, Xiaofeng Niu, Lijuan Deng, Depei Gao, Qinqing Li, Jun Yang

**Affiliations:** 1 Department of Radiology, The Third Affiliated Hospital of Kunming Medical University, Yunnan Cancer Hospital, Peking University Cancer Hospital Yunnan, Kunming, China; 2 Department of Pathology, The Third Affiliated Hospital of Kunming Medical University, Yunnan Cancer Hospital, Peking University Cancer Hospital Yunnan, Kunming, China

**Keywords:** Breast cancer, Neoadjuvant chemotherapy, Miller–Payne system, MRI, Radiomics

## Abstract

**Background:**

Breast cancer’s personalized management requires better risk stratification. Recent studies focus on differentiating the pathological complete response (pCR) from non-pCR, which lacks accuracy in prognostic prediction and therapy guidance for most non-pCR patients. We aimed to better stratify neoadjuvant chemotherapy (NAC) response and early identification of poor responders in the non-pCR population.

**Methods:**

Pretreatment MRI scans were obtained retrospectively from breast cancer patients who had NAC followed by surgery (January 2021-October 2023). Pathological response to NAC was assessed using the Miller-Payne (MP) grading system, with grades 1–2 indicating poor response and grades 3–5 indicating good response. Logistic regression was used to identify variables associated with MP grading and to build predictive models based on the radiomics score, clinicopathological features, and their combination. Additionally, machine learning models were also trained. The models were assessed for discrimination, calibration, and decision-making ability. Shapley Additive Explanations (SHAP) analysis was specifically performed to interpret the final machine learning model.

**Results:**

A total of 336 patients were included (mean age, 48.75 ± 9.52 years; training set, 235; test set, 101). Radiomics score (OR = 1.46, 95% CI: 1.09, 1.99; *P* = 0.013) and human epidermal growth factor receptor 2 (HER2) status (OR = 5.93, 95% CI: 2.58, 16.16; *P* < 0.001) were independently associated with MP grades. The logistic regression, XGBoost, and decision tree combined models demonstrated enhanced discrimination performance, with area under the receiver operating characteristic curve (AUC) of 0.77 (95% CI: 0.67, 0.87), 0.74 (95% CI: 0.65, 0.84), and 0.71(95% CI: 0.59, 0.82), respectively.

**Conclusions:**

The combined model integrating pretreatment MRI radiomics score and HER2 status effectively differentiated between MP grades 1–2 and 3–5 in breast cancer following NAC. The study improved response stratification, with a specific emphasis on early detection of poor NAC responders in order to provide precise prognostic guidance and influence treatment options for this patient population.

**Trial registration:**

Not applicable.

**Supplementary Information:**

The online version contains supplementary material available at 10.1186/s40644-026-00990-5.

## Introduction

Breast cancer is one of the most common malignant tumors in women and accounts for the second leading cause of female cancer mortality [1]. Neoadjuvant chemotherapy (NAC) is used to shrink lesions, facilitate surgery, and improve patient prognosis in locally advanced or large breast cancers [2–4]. The assessment of tumor response to NAC is subsequently used to predict prognosis. Pathological complete response (pCR) is associated with improved disease-free survival (DFS) and overall survival (OS) [5]. However, NAC response varies widely, with only approximately 19% of patients achieving pCR and 8%-30% showing insensitivity [6, 7]. Recent studies have focused on pCR/non-pCR classification for treatment personalization [6, 8, 9]. However, this overlooks the response heterogeneity within the larger non-pCR population, which includes varying pathological responses from no/poor to improved response, with poorer responses correlating with adverse outcomes [10]. This limitation undermines both therapeutic guidance and prognostic accuracy for non-pCR patients, who constitute a critical subgroup for improving overall outcomes in breast cancer. Therefore, more refined stratification of patients failing to achieve pCR is clinically imperative.

Breast cancer NAC response is assessed clinically using Response Evaluation Criteria in Solid Tumors (RECIST) 1.1 and pathologically via post-surgical tumor and lymph node evaluation, the gold standard [11]. The Miller-Payne (MP) grading system compares post-treatment surgical specimens to pre-treatment diagnostic biopsy specimens, grading the loss of tumor cells from 1 to 5, with higher grades indicating better response [12]. Romero et al. reported moderate agreement between the MP grading system and RECIST criteria, but MP grades demonstrated superior correlation with both OS and recurrence-free survival (RFS) compared to RECIST [13]. In addition, several studies have also revealed a significant correlation between MP grades and DFS and OS, and MP grade was an independent predictor of DFS/OS, lower MP grade correlates with adverse prognosis [12–15]. The MP grading system defines grades 1–2 as no change or less than 30% reduction in tumor cellularity. In the study, we classified patients with MP grades 1–2 as “poor response”, who derived minimal benefit from NAC and demonstrated unfavorable long-term prognosis. In contrast, patients with MP grades 3–5 were categorized as “good response”, showing varying degrees of NAC benefit with relatively better outcomes. This classification facilitates focusing therapeutic intensification on poor responders and better identifying optimal candidates for NAC.

Breast MRI plays a crucial role in breast cancer diagnosis and NAC response prediction due to its radiation-free, high soft-tissue resolution, and multimodal imaging capabilities [16]. With the advancement of artificial intelligence, breast MRI radiomics has made significant progress. It facilitates the extraction, analysis, and quantification of high-throughput imaging features that are imperceptible to the human eye, thereby offering more comprehensive tumor information [17, 18]. Several studies have demonstrated that MRI radiomics features can serve as effective predictors of NAC response in breast cancer [19–21].

This study aims to develop an early and non-invasive predictive model for pathological MP grading based on pretreatment MRI radiomics features, which may enhance personalized management strategies for breast cancer patients.

## Methods

### Study sample

This retrospective study was approved by the Institutional Ethics Committee and informed consent was waived for all enrolled patients (Approval No. KYLX2025-022). The study was designed according to the Transparent Reporting of a Multivariable Prediction Model for Individual Prognosis or Diagnosis (TRIPOD) guidelines [22], and radiomics procedures followed the Radiomics Quality Score (RQS) checklist [17].

**Fig. 1:**
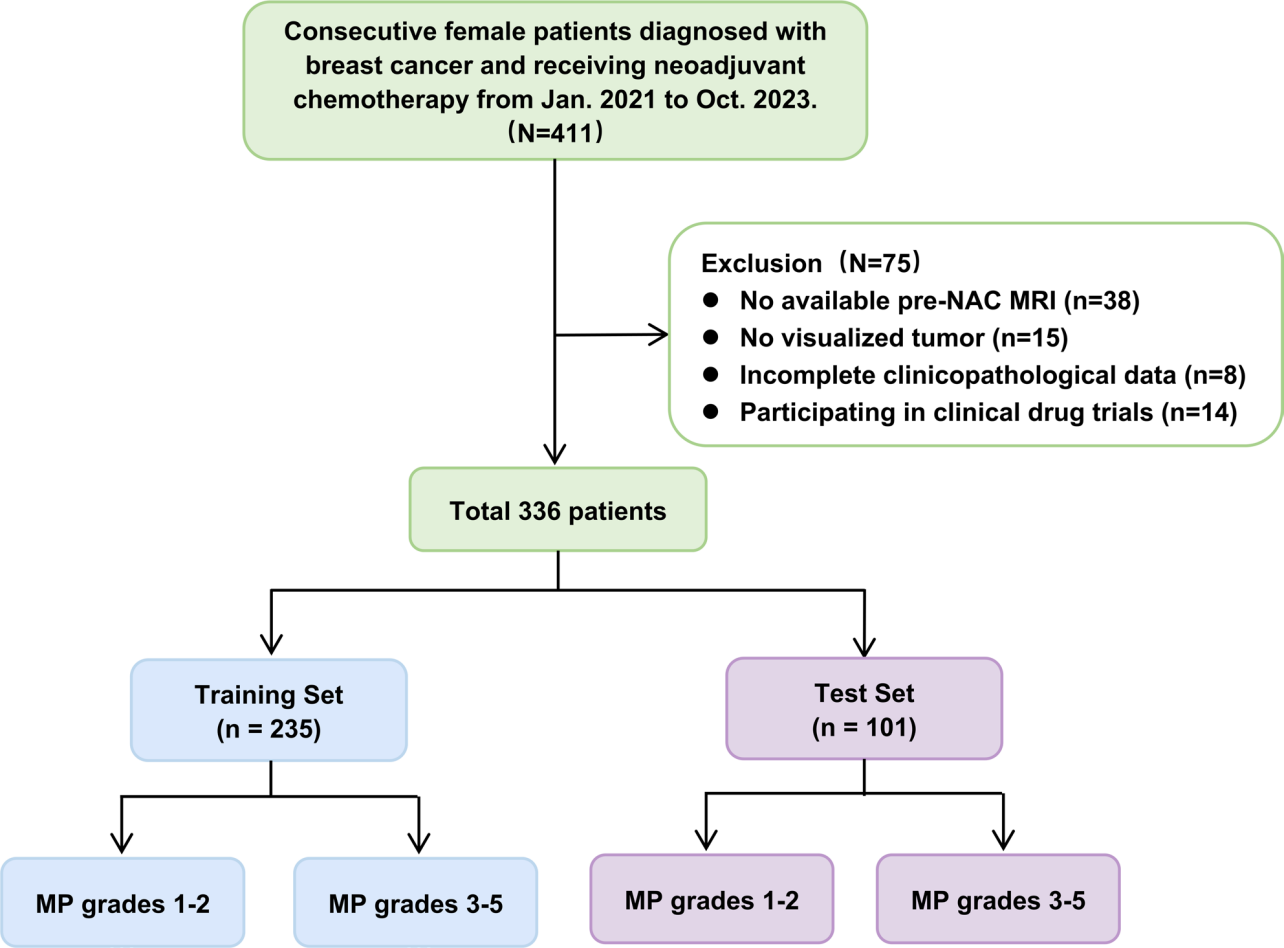
Patients selection flowchart. MP = Miller-Payne

Female patients with pathologically confirmed invasive breast cancer who underwent NAC followed by surgery between January 2021 and October 2023 were included in this study. Clinicopathologic data were retrospectively collected from the medical record system for each patient. The inclusion criteria were as follows: (1) pathologically diagnosed invasive breast cancer before treatment; (2) completion of a full NAC cycle; (3) radical surgery following NAC, with treatment response assessed using the MP grading system. The following exclusion criteria were applied: (1) no available MRI scans within two weeks before NAC or poor image quality, (2) no tumor visibility on MRI images, (3) incomplete clinicopathological data, and (4) participating in clinical drug trials (Response heterogeneity due to differences in treatment protocols). Finally, 336 breast tumors were included in the study cohort. The patient selection flowchart is shown in Fig. 1.

### Pathological examination and Miller-Payne grading system

Prior to NAC, ultrasound-guided core needle biopsies were systematically obtained from all breast tumor lesions for comprehensive histopathological evaluation. The biopsy specimens were subjected to standardized immunohistochemical (IHC) analyses to determine tumor classification, including estrogen receptor (ER), progesterone receptor (PR), human epidermal growth factor receptor 2 (HER2) status (assessed by IHC and fluorescence in situ hybridization when equivocal), and Ki-67 proliferation index. Postoperative specimens were evaluated independently by two board-certified pathologists blinded to clinical outcomes, with consensus review for discordant cases, and the pathological response to NAC was assessed using the MP grading system [12]:

- Grade 1: No change or some alteration to individual malignant cells but no reduction in overall cellularity.
- Grade 2: A minor loss of tumor cells but overall cellularity still high; up to 30% loss.
- Grade 3: Between an estimated 30% and 90% reduction in tumor cells.
- Grade 4: A marked disappearance of tumor cells such that only small clusters or widely dispersed individual cells remain; more than 90% loss of tumor cells.
- Grade 5: No malignant cells identifiable in sections from the site of the tumor; only vascular fibroelastotic stroma remains often containing macrophages. However, ductal carcinoma in situ (DCIS) may be present.

In this study, grades 1–2 was categorized as poor response, and grades 3–5 was categorized as good response.

### MRI image acquisition and radiomics features extraction

All pretreatment MRI scans were acquired using a Siemens 1.5T MRI scanner at our institution. The pulse sequences, contrast injection protocols, and data acquisition times were detailed in Table S1. Subtraction images derived from the first post-contrast phase were utilized for subsequent analysis.

The region of interest (ROI) was semi-automatically segmented by radiologist A (with 2 years of breast MRI experience) using 3D Slicer’s level tracing algorithm (LTA, version 5.6.2; www.slicer.org). LTA, a variant of active contour methods, is particularly suitable for breast tumor delineation on contrast-enhanced T1-weighted MRI as it primarily leverages image intensity gradients and contrast differences to delineate tumor boundaries. The algorithm’s built-in smoothness constraints further ensure continuous, biologically plausible tumor boundaries, effectively minimizing erroneous segmentation from image noise or artifacts, which is crucial for delineating breast tumors with complex and irregular morphologies. As a semi-automatic method, LTA requires the user to define an initial seed point or a rough contour within the tumor to accurately guide its initiation and focus on the target region. Our segmentation criteria for tumors on contrast-enhanced T1 MRI primarily included: delineating only the largest lesion in cases of multiple lesions; careful exclusion of normal enhancing glandular tissue or vessels; and exclusion of non-enhancing necrotic or cystic components within the tumor. To evaluate intra-observer and inter-observer delineation consistency, radiologist A (three months after initial segmentation) and radiologist B (with 8 years of breast MRI experience) independently re-segmented the tumors of 48 randomly selected patients using the same method. A total of 107 radiomics features compliant with the Image Biomarker Standardization Initiative (IBSI) [23] were extracted per segmented volume from all 336 volumes using PyRadiomics (version 3.0). These included 18 first-order, 14 shape, and 75 texture features (Table S2).

### Features selection and radiomics score deriving

The inter-observer and intra-observer consistency of tumor segmentation was assessed using the intraclass correlation coefficient (ICC). The interpretation of ICC values was based on the following criteria: ICC < 0.50 indicated poor consistency; 0.50–0.75 indicated moderate consistency; 0.75–0.90 indicated good consistency; and > 0.90 indicated excellent consistency. In this study, radiomics features demonstrating an intra- or inter-observer ICC below 0.75 were subsequently excluded. The radiomics features extracted from radiologist A’s segmentations were utilized for all subsequent analyses. Features associated with MP grading were screened using Student’s *t* test or the Mann-Whitney *U* test, and those with *P*-values less than 0.05 were retained. Spearman correlation analysis was subsequently performed, and features with correlation coefficients exceeding 0.8 were clustered into a single representative feature to minimize multicollinearity. Finally, the least absolute shrinkage and selection operator (LASSO) algorithm combined with 10-fold cross-validation was applied to select the optimal radiomics features and their corresponding weight coefficients. Radiomics scores were computed via coefficient-weighted linear combination (1) and Z-score normalized.

1 $$\begin{aligned}\:\mathrm{R}\mathrm{a}\mathrm{d}\mathrm{i}\mathrm{o}\mathrm{m}\mathrm{i}\mathrm{c}\mathrm{s}\:\mathrm{s}\mathrm{c}\mathrm{o}\mathrm{r}\mathrm{e}&={\beta}_{0}+{\beta}_{1}\mathrm{*}{F}_{1}+{\beta}_{2}\mathrm{*}{F}_{2}\cr&\quad+\dots+{\beta}_{n}\mathrm{*}{F}_{n}\end{aligned}$$

where *F*_*n*_ is the feature value, *β*_*n*_ is its coefficient, and *β*_*0*_ is the linear regression equation’s intercept.

### Predictive model construction

**Fig. 2:**
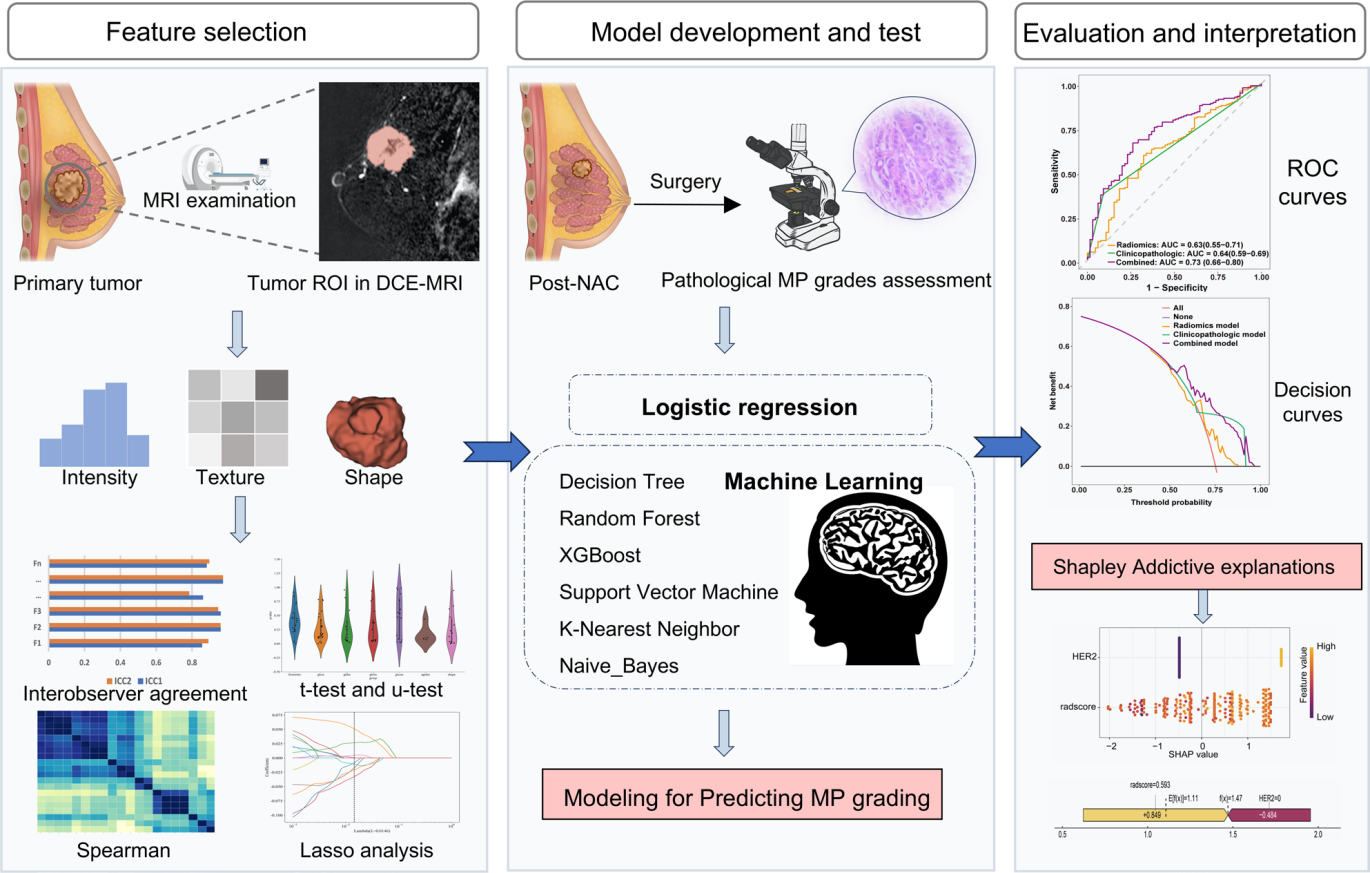
Radiomics flowchart for predicting MP grading after NAC for breast cancer. DCE = dynamic contrast-enhanced. NAC = neoadjuvant chemotherapy. MP = Miller-Payne. ROC = receiver operating characteristic curve

Randomization seeds were used to divide the study population into training (*n* = 235) and test (*n* = 101) sets at a 7:3 ratio. Between-group comparisons confirmed comparable distributions of significant characteristics between the two sets. Univariate logistic regression identified variables associated with MP grading, and those with *P* < 0.05 were included in a multivariate logistic regression model using backward stepwise selection to determine independent predictors of MP grading. Three predictive models were developed using logistic regression: (1) a radiomics model based on the radiomics score; (2) a clinicopathologic model using clinicopathologic features; and (3) a combined model incorporating both the radiomics score and clinicopathologic features. Additionally, six machine learning models—decision tree, random forest, XGBoost, support vector machine, K-Nearest Neighbor, and naive bayes—were developed based on the combined features. Receiver operating characteristic (ROC) curves, calibration curves, and decision curves were plotted to evaluate model performance. Shapley Additive Explanation (SHAP) analysis was employed to interpret the XGBoost model. Figure 2 illustrates the study workflow.

### Statistical analysis

Continuous variables were expressed as mean ± standard deviation (SD) or median (interquartile range, IQR). Categorical variables were presented as counts (percentages). For continuous variables, inter-group comparisons were performed using the Student’s *t*-test if they met the assumptions of normal distribution and homogeneity of variances; otherwise, the Wilcoxon-Mann-Whitney *U* test was used. For categorical variables, inter-group comparisons were conducted using the Pearson χ² test, with Fisher’s exact test applied when more than 20% of the cells had an expected count less than 5. Univariate logistic regression analysis was employed to identify variables associated with MP grading. Multivariate logistic regression analysis was used to determine independent predictors of MP grading. The performance of the models was evaluated using area under the receiver operating characteristic curve (AUC), calibration curves, and decision curve analysis (DCA). Statistical analyses were conducted using R software (version 4.4.2). All statistical tests were two-sided, and a *P* < 0.05 was considered statistically significant.

## Results

### Study dataset characteristics

Among the 411 initially enrolled patients, 75 were excluded: 38 due to the absence of pretreatment MRI scans or poor image quality, 15 due to no visible tumors, 8 due to incomplete clinicopathological data, and 14 due to participation in clinical trials, as illustrated in Fig. 1. The remaining 336 patients included 90 with MP grades 1–2 and 246 with MP grades 3–5. The training set comprised 235 patients (mean age: 49.06 ± 9.89 years), and the test set included 101 patients (mean age: 48.05 ± 8.62 years). Baseline characteristics are shown in Table 1. No significant differences were observed in key variables between the training and test sets.

**Table 1:** The clinicopathologic characteristics of training and test set

Characteristic	Training Set (*n* = 235)	Test Set (*n* = 101)	*P* Value
Age (Mean ± SD)	49.06 ± 9.89	48.05 ± 8.62	0.35
HR status (%)			0.754
Negative	47 (20)	18 (18)	
Positive	188 (80)	83 (82)	
HER2 status (%)			1
Negative	165 (70)	71 (70)	
Positive	70 (30)	30 (30)	
Ki67 index (%)			0.102
< 20%	47 (20)	12 (12)	
≥ 20%	188 (80)	89 (88)	
Molecular subtype (n%)			0.849
HR+/HER2-	143 (61)	61 (60)	
HR+/HER2+	45 (19)	22 (22)	
HR-/HER2+	25 (11)	8 (8)	
HR-/HER2-	22 (9)	10 (10)	
Clinical T stage (%)			0.537
T1-T2	161 (69)	65 (64)	
T3-T4	74 (31)	36 (36)	
Clinical N stage (%)			0.007
N0	36 (15)	9 (9)	
N1	150 (64)	55 (54)	
N2-N3	49 (21)	37 (37)	
Treatment response			0.676
MP grades 1–2	65 (28)	25 (25)	
MP grades 3–5	170 (72)	76 (75)	

Notes: HR = hormone receptor, HER2 = human epidermal growth factor receptor 2, MP = Miller–Payne. Categorical data are expressed as the number of patients, with percentages in parentheses. Categorical variables’ P values were obtained using the χ² or Fisher exact tests. Continuous data are expressed as mean ± SD. p-values were calculated use Wilcoxon-Mann-Whitney or student t test

### Feature selection and radiomics score

A total of 104 radiomics features with inter- and intra-observer correlation coefficients ≥ 0.75 were included in subsequent selection (Fig S1). Student’s *t* test or Mann-Whitney *U* test retained 20 features significantly associated with MP grading (*P* < 0.05) (Fig S2). Spearman correlation analysis identified 14 uncorrelated features (Fig S3). LASSO selected 9 non-zero coefficient features, and the radiomics score for each tumor was calculated using Eq. (1) (Table S3; Fig S4-5).

### Evaluation of model performance

**Fig. 3:**
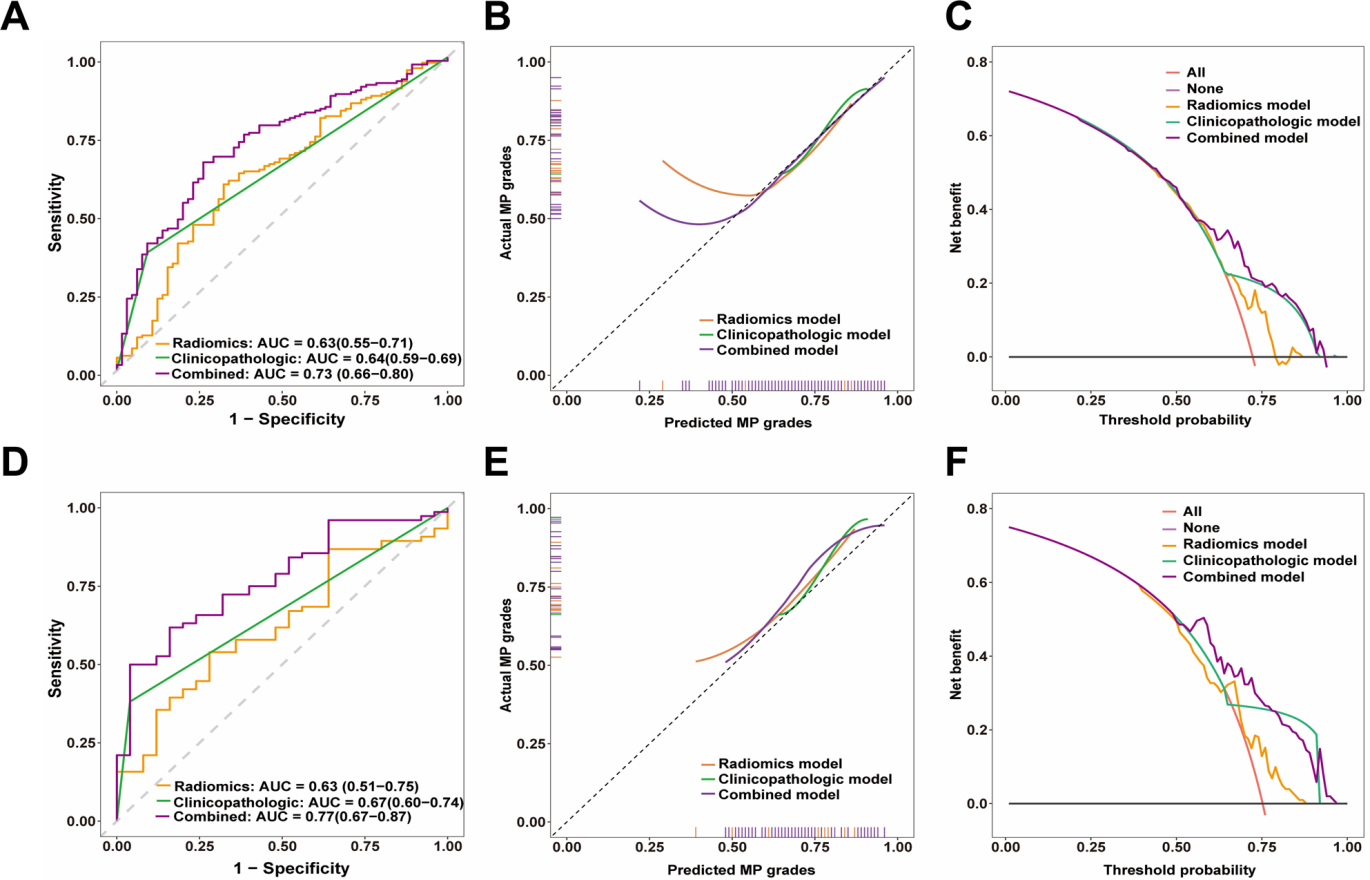
Receiver operating characteristic curves, calibration curves, and decision curves of three logistic regression models for predicting MP grading in the (**A**, **B**, **C**) training set and (**D**, **E**, **F**) test set. Calibration curves were plotted using bootstrapping with 1000 resamples. AUC = area under receiver operating characteristic curve. MP = Miller-Payne

Univariate logistic regression revealed that HER2 status (OR, 5.94 [95% CI: 2.60, 16.07]; *P* < 0.001), molecular subtype (OR range, 1.22–6.57; *P* < 0.68), and radiomics score (OR, 1.46 [95% CI: 1.10–1.96]; *P* = 0.009) were significantly associated with MP grading in the training set (Table 2). Backward stepwise regression identified HER2 status (OR = 5.93, 95% CI: 2.58, 16.16; *P* < 0.001) and radiomics score (OR, 1.46 [95% CI: 1.09–1.99]; *P* = 0.013) as independent predictors of MP grading (Table 3). The logistic regression model based on the radiomics score achieved an AUC of 0.63 (95% CI: 0.51, 0.75) in the test set, while the clinicopathologic model (based on HER2 status) achieved an AUC of 0.67 (95% CI: 0.60, 0.74). The combined model demonstrated significantly improved predictive performance, with AUC of 0.77 (95% CI: 0.67, 0.87) in the test set. The calibration curve analysis demonstrated good agreement between the predicted and actual values. Figure 3 illustrates the ROC curves, calibration curves, and decision curves of the logistic regression models.

**Table 2:** Univariable logistic regression analysis for the association between MP grades to NAC and patient characteristics

**Characteristics**	Training Set (*n* = 235)		
	**OR**	**95%CI**	**P Value**
Age	0.991	0.963–1.02	0.551
HR status (positive vs. negative)	0.558	0.24–1.186	0.149
HER2 status (positive vs. negative)	5.937	2.603–16.07	< 0.001
Ki67 index (≥20%vs < 20%)	1.294	0.634–2.557	0.467
Molecular subtype			
HR+/HER2 + vs. HR+/HER2-	5.857	2.208–20.29	0.001
HR-/HER2 + vs. HR+/HER2-	6.571	1.842–41.98	0.013
HR-/HER2- vs. HR+/HER2-	1.224	0.483–3.384	0.679
Clinical T stage(T3-T4 vs. T1-T2)	0.712	0.391–1.31	0.269
Clinical N stage			
N1 vs. N0	0.76	0.303–1.737	0.533
N2-N3 vs. N0	0.589	0.211–1.55	0.294
Radiomics score	1.460	1.014–1.958	0.009

Notes: HR = hormone receptor, HER2 = human epidermal growth factor receptor 2, MP = Miller–Payne

**Table 3:** Multivariable logistic regression analysis by using backward stepwise selection patient variables for their association with MP grades

Set (*n* = 235)	Training		
**Characteristics**	**OR**	**95%CI**	**P Value**
Intercept	0.209	0.035–1.127	0.074
HER2 status (positive vs. negative)	5.933	2.581–16.16	< 0.001
Radiomics score	1.459	1.090–1.986	0.013

Notes: HER2 = human epidermal growth factor receptor 2

**Fig. 4:**
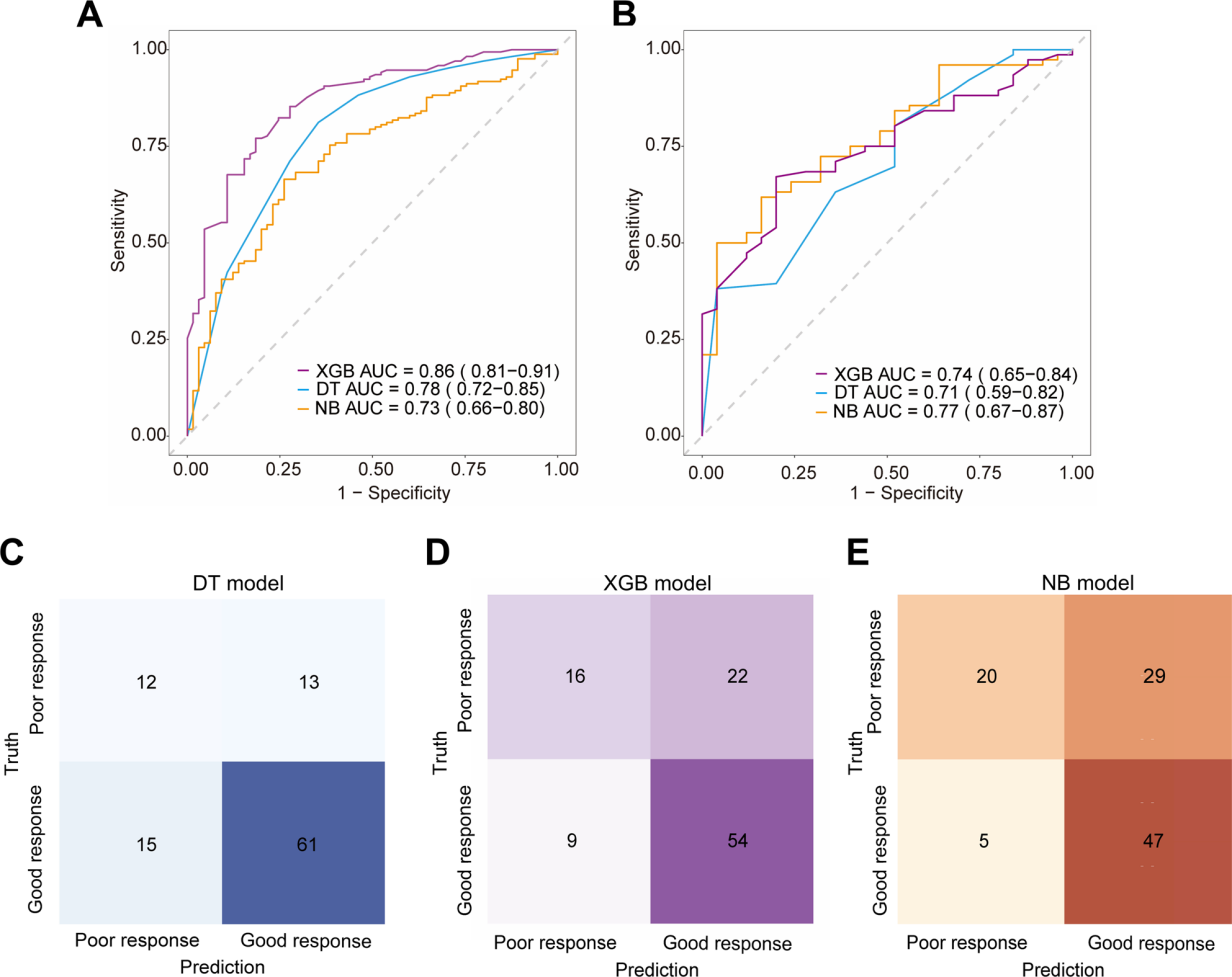
Receiver operating characteristic curves of the three machine learning models based on combined features: (**A**) training set and (**B**) test set. (**C**, **D**, **E**) Confusion matrices of the three machine learning models in the test set. DT = Decision Tree, XGB = XGBoost, NB = Naive Bayes. AUC = area under the receiver operating characteristic curve

Among six machine learning models based on combined features, XGBoost, decision tree, and naive bayes performed well. The naive bayes model demonstrated performance comparable to logistic regression, while XGBoost and decision tree achieved AUCs of 0.86 (95% CI: 0.81, 0.91) and 0.78 (95% CI: 0.72, 0.85) in the training set, 0.74 (95% CI: 0.65, 0.84) and 0.71 (95% CI: 0.59, 0.82) in the test set, respectively. Figure 4 illustrates the ROC curves and confusion matrices of the top three performing machine learning models. Sensitivity, specificity, positive predictive value, negative predictive value, and F1 score are detailed in (Table S4).

### Clinical utility and model interpretation

Decision curve analysis (Fig. 3C, F) of the three logistic regression models based on the radiomics score, clinicopathologic variables, and combined features demonstrated that the combined model outperformed strategies assuming all patients were positive or negative, as well as models based solely on the radiomics score or HER2 status. In particular, the combined model has excellent clinical utility in predicting pathologic MP grading when considering a wide range of threshold probabilities of 60% or higher, and can provide effective support for clinical decision-making. Although being less than the combined model’s net benefit, the clinicopathologic and radiomics models nonetheless showed some clinical utility at certain thresholds.

**Fig. 5:**
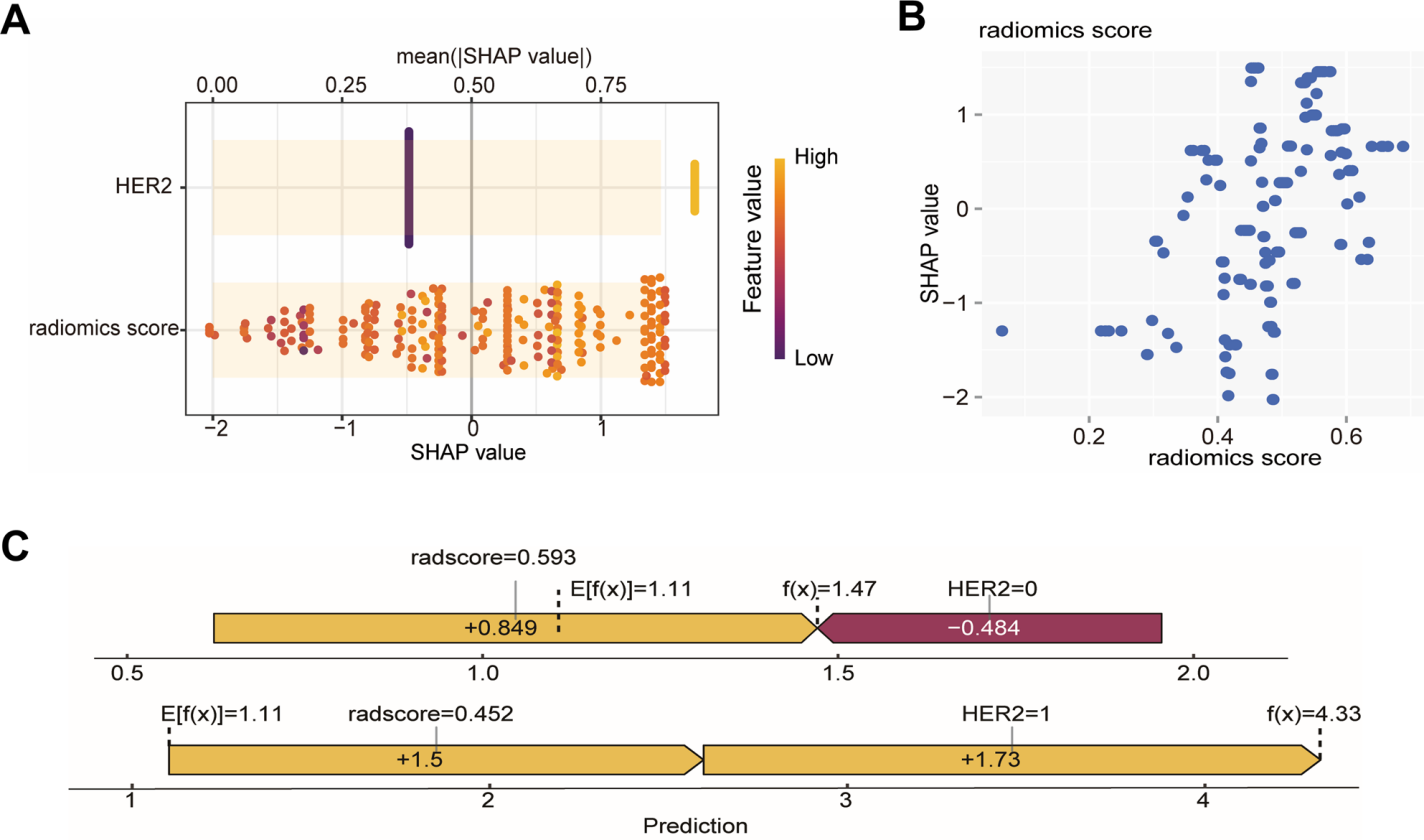
SHAP summary plot for the XGBoost model. (**A**) Feature importance ranking (bar chart) and SHAP values distribution for each sample across different features (scatter plot). Higher feature values are indicated by yellow dots, and lower eigenvalues are indicated by purple dots. (**B**) SHAP dependency plot of radiomics scores. SHAP values < 0 mainly corresponded to radiomics score features < 0, while SHAP values > 0 were primarily linked to features > 0. (**C**) Interpretability analysis of 2 independent samples. Features are shown as arrows; right-pointing arrows signify positive effects, left-pointing arrows indicate negative effects, and arrow length reflects the degree of influence. MP = Miller-Payne. SHAP = shapley addictive explanations

**Fig. 6:**
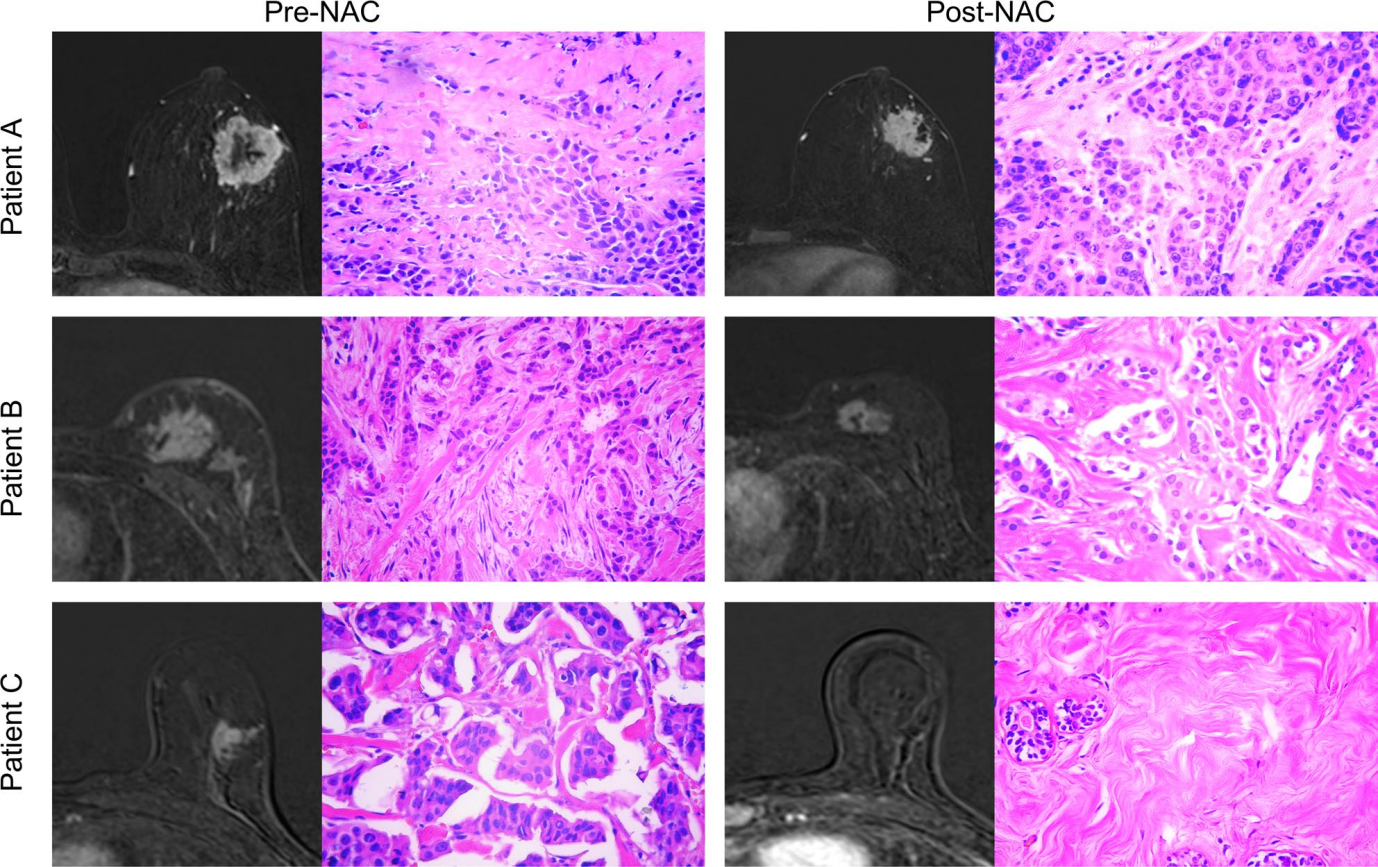
Examples of DCE-MRI subtraction images and histopathological sections (H&E, 20×) from different breast tumors, showing responses before and after NAC. Patient (**A**) A 55-year-old female with HER2-negative and a low radiomics score (-1.80) showed minimal tumor shrinkage after NAC, achieving MP grade 2 on postoperative pathology. Patient (**B**) A 46-year-old female with HER2-negative and a moderate radiomics score (0.24) exhibited moderate tumor regression, with MP grade 3 on postoperative pathology. Patient (**C**) A 53-year-old female with HER2-positive and a high radiomics score (1.38) had no visible tumor after NAC, with MP grade 5 on postoperative pathology. DCE = dynamic contrast-enhanced. NAC = neoadjuvant chemotherapy. MP = Miller-Payne

This study conducted a SHAP analysis on the XGBoost model to evaluate features importance and their predictive roles. Figure 5 presents the SHAP summary analysis of the XGBoost model. Results showed HER2 status had a slightly higher SHAP value than the radiomics score, with little difference, indicating both are key factors for MP grading prediction. HER2 negativity contributed negatively to the prediction outcome, while HER2 positivity contributed positively, meaning HER2-negative patients were more likely to be classified as MP grades 1–2, and HER2-positive patients were more likely to be classified as MP grades 3–5. Tumors with low radiomics scores (cut-off = 0.053; Table S5) were significantly associated with MP grades 1–2, while those with high scores correlated with MP grades 3–5. Additionally, low radiomics score tumors exhibited a higher proliferation index (Ki67) and more advanced clinical T/N stage than high radiomics score tumors (Table S6). Representative examples of MP grading across varying radiomics scores are illustrated in Fig. 6.

## Discussion

The MP grading system reflects treatment efficacy across five grades [12–14]. Patients with MP grades 1–2 derive little to no benefit from NAC and may even miss the optimal surgical window due to NAC, potentially delaying disease. Personalized management of these patients remains a critical clinical challenge [24]. In contrast, patients with MP grades 3–5 benefit to varying degrees from NAC, indicating its value for this group. While current research predominantly focuses on the pCR group, the poor responders (MP grades 1–2) accounting for a significant proportion has been largely overlooked [5, 25, 26]. To address this gap, this study aimed to develop an MP grading prediction model based on readily available imaging and clinicopathological data, particularly to identify non-responders or poor responders (MP grades 1–2), with the goal of guiding clinical decision-making and providing additional prognostic information.

In this study cohort, 27% (90/336) of patients showed a poor response (MP grades 1–2), whereas 73% (246/336) achieved a good response (MP grades 3–5). The pCR rate (MP grade 5) was 28% (95/336). Notably, the proportion of poor responders is comparable to the pCR rate, further underscoring the critical importance of these patients in improving the overall prognosis of breast cancer patients. Logistic regression analysis revealed that the radiomics score and HER2 status are independent predictors of MP grading. The combined logistic regression model, naive bayes model, XGBoost model, and decision tree model demonstrated strong predictive performance for MP grading. The naive bayes model and logistic regression model exhibited comparable performance, which may be attributed to weak correlations among features and balanced contributions to the prediction target, or strong linear separability of the data. Compared to the logistic regression model, other machine learning models did not show significantly improved discriminative ability. This may be due to the limited number of features included in this study and the weak interactions among features. In the interpretability analysis of the XGBoost model, the radiomics score and HER2 status were identified as core predictors of MP grading. HER2-negative patients were more likely to be predicted as MP grades 1–2, whereas HER2-positive patients tended to be predicted as MP grades 3–5, consistent with previous studies [27] and clinical observations that HER2-positive patients benefit from targeted therapy, thereby improving the efficacy of NAC [28, 29]. Dependency analysis of the radiomics score revealed a positive correlation with treatment response, which is consistent with the findings reported by Ramtohul et al. [30]. In summary, the combined model incorporating the radiomics score and HER2 status demonstrated strong discriminative ability and clinical utility in predicting MP grading.

A previous study effectively predicted NAC-insensitive patients (MP 1–2) using MRI radiomics combined with clinical features [27], compared to that study, our research expanded the sample size, incorporated machine learning models and performed interpretability analysis, thereby enhancing the model’s clinical utility. These findings suggest that radiomics can effectively predict MP grading, consistent with its successful application in predicting pCR [8, 30–32]. Radiomics analysis of non-invasive imaging may provide high-throughput and comprehensive information reflecting tumor biological behavior, enabling early prediction of treatment response. In this study, multiple radiomics features were integrated into a single composite metric, termed the radiomics score. This approach reduces feature dimensionality, mitigates overfitting from high-dimensional data, and simplifies the model for clinical application. Additionally, the composite score intuitively reflects the collective contribution of all radiomics features to predictive outcomes, making it suitable for patient stratification and clinical decision-making. Several studies have increasingly adopted radiomics scores to replace complex multi-feature analyses [30, 32, 33], demonstrating their potential for clinical utility. Furthermore, the advancement of artificial intelligence has significantly propelled the development of radiomics. For instance, several studies have employed radiomics-based machine learning models to predict pCR in breast cancer, often outperforming traditional linear models, with reported AUC values ranging from 0.71 to 0.87 [34–38]. However, while these studies have primarily concentrated on pCR prediction, research on more sophisticated response evaluation systems such as MP grading remains limited, representing a promising direction for future investigation.

This study has several limitations. First, as a single-center retrospective study, it lacks external validation, which may limit the generalizability of the findings and introduce potential selection bias in the study population. Second, due to insufficient follow-up data, we were unable to evaluate RFS and OS based on radiomics scores, resulting in a gap in our understanding of long-term prognosis. Third, the predictive accuracy of the models could potentially be improved by stratifying the study cohort into distinct subgroups based on breast cancer subtypes [7, 9] and developing subtype-specific prediction models. Finally, incorporating multimodal functional imaging data, such as histopathological features and ultrasound characteristics [39, 40], may further enhance model performance and provide a more comprehensive assessment of treatment response.

## Conclusions

Following NAC, pathological MP grading in breast cancer patients was accurately predicted using radiomics score derived from pretreatment dynamic contrast-enhanced MRI combined with HER2 status. These findings suggest the potential of radiomics as a biomarker for drug sensitivity in future research. Future studies should focus on developing a multiclassification model that better aligns with actual pathological grading and exploring alternative treatment strategies tailored for patients with poor treatment responses.

## Supplementary Information

Below is the link to the electronic supplementary material.

Supplementary Material 1

## Data Availability

The datasets used and/or analysed during the current study are available from the corresponding author on reasonable request.

## Abbreviations

MP: Miller-Payne
NAC: Neoadjuvant chemotherapy
AUC: Area under the receiver operating characteristic curve
