# Supplementary material for: Pretreatment MRI radiomics for predicting pathological Miller-Payne grading in breast cancer following neoadjuvant chemotherapy
Source: Cancer Imaging. 2026 Jan 16;26:25. doi: 10.1186/s40644-026-00990-5 (PMC12892561; doi:10.1186/s40644-026-00990-5)

**Supplemental Tabes**

**Table S1**

**Breast DCE-MRI Scanning Parameters.**

| Parameters | Details |
| --- | --- |
| Scanner | Siemens 1.5T Avanto |
| Sequence | 3D Gradient echo |
| Repetition Time (ms) | 4.4 |
| Echo Time (ms) | 1.4 |
| Field of view(mm) | 400×400 |
| Acquisition Matrix (n) | 360×360 |
| Voxel Size(mm) | 1.0 |
| Slice Thickness (mm) | 1 |
| Flip angle | 10° |
| Contrast agent | Gadopentetate Dimeglumine, Gadodiamide |
| Injection protocols | 0.2ml/kg, 2ml/s |

Image Acquisition and Processing: Pre-contrast T1-weighted baseline images were acquired prior to contrast agent administration. Dynamic post-contrast T1-weighted imaging was initiated at 90 seconds after injection, with five consecutive phases acquired. Subtraction images were generated by subtracting the pre-contrast baseline from each post-contrast phase for further analysis.

**Table S2**

**List of Radiomics Features Extracted from Pyradiomics.**

| Category | Features |
| --- | --- |
| firstorder features | 1.10Percentile |
|  | 2.90Percentile |
|  | 3.Energy |
|  | 4.Entropy |
|  | 5.InterquartileRange |
|  | 6.Kurtosis |
|  | 7.Maximum |
|  | 8.Mean |
|  | 9.MeanAbsoluteDeviation |
|  | 10.Median |
|  | 11.Minimum |
|  | 12.Range |
|  | 13.RobustMeanAbsoluteDeviation |
|  | 14.RootMeanSquared |
|  | 15.Skewness |
|  | 16.TotalEnergy |
|  | 17.Uniformity |
|  | 18.Variance |
| Shape-based | 19. Elongation |
|  | 20. Flatness |
|  | 21. LeastAxisLength |
|  | 22. MajorAxisLength |
|  | 23. Maximum2DDiameterColumn |
|  | 24. Maximum2DDiameterRow |
|  | 25. Maximum2DDiameterSlice |
|  | 26. Maximum3DDiameter |
|  | 27. MeshVolume |
|  | 28. MinorAxisLength |
|  | 29. Sphericity |
|  | 30. SurfaceArea |
|  | 31. SurfaceVolumeRatio |
|  | 32. VoxelVolume |
| Gray Level Co-occurrence Matrix (GLCM) | 33. Autocorrelation  34. ClusterProminence  35. ClusterShade  36. ClusterTendency  37. Contrast  38. Correlation  39. DifferenceAverage  40. DifferenceEntropy  41. DifferenceVariance  42. Id  43. Idm  44. Idmn  45. Idn  46. Imc1  47. Imc2  48. InverseVariance  49. JointAverage  50. JointEnergy  51. JointEntropy  52. MCC  53.MaximumProbability  54.SumAverage  55.SumEntropy  56.SumSquares |
| Gray Level Dependence Matr (GLDM) | 57. DependenceEntropy |
|  | 58. DependenceNonUniformity |
|  | 59. DependenceNonUniformityNormalized |
|  | 60. DependenceVariance |
|  | 61. GrayLevelNonUniformity |
|  | 62. GrayLevelVariance |
|  | 63. HighGrayLevelEmphasis |
|  | 64. LargeDependenceEmphasis |
|  | 65. LargeDependenceHighGrayLevelEmphasis |
|  | 66. LargeDependenceLowGrayLevelEmphasis |
|  | 67. LowGrayLevelEmphasis |
|  | 68. SmallDependenceEmphasis |
|  | 69. SmallDependenceHighGrayLevelEmphasis |
|  | 70. SmallDependenceLowGrayLevelEmphasis |
| Gray Level Run Length Matrix   (GLRLM) | 71. GrayLevelNonUniformity |
|  | 72. GrayLevelNonUniformityNormalized |
|  | 73. GrayLevelVariance |
|  | 74. HighGrayLevelRunEmphasis |
|  | 75. LongRunEmphasis |
|  | 76. LongRunHighGrayLevelEmphasis |
|  | 77. LongRunLowGrayLevelEmphasis |
|  | 78. LowGrayLevelRunEmphasis |
|  | 79. RunEntropy |
|  | 80. RunLengthNonUniformity |
|  | 81. RunLengthNonUniformityNormalized |
|  | 82. RunPercentage |
|  | 83. RunVariance |
|  | 84. ShortRunEmphasis |
|  | 85. ShortRunHighGrayLevelEmphasis |
|  | 86. ShortRunLowGrayLevelEmphasis |
| Gray Level Size Zone Matrix  (GLSZM) | 87. GrayLevelNonUniformity |
|  | 88. GrayLevelNonUniformityNormalized |
|  | 89. GrayLevelVariance |
|  | 90. HighGrayLevelZoneEmphasis |
|  | 91. LargeAreaEmphasis |
|  | 92. LargeAreaHighGrayLevelEmphasis |
|  | 93. LargeAreaLowGrayLevelEmphasis |
|  | 94. LowGrayLevelZoneEmphasis |
|  | 95. SizeZoneNonUniformity |
|  | 96. SizeZoneNonUniformityNormalized |
|  | 97. SmallAreaEmphasis |
|  | 98. SmallAreaHighGrayLevelEmphasis |
|  | 99. SmallAreaLowGrayLevelEmphasis |
|  | 100. ZoneEntropy |
|  | 101. ZonePercentage |
|  | 102. ZoneVariance |
| Neighboring Gray Tone Difference Matrix (NGTDM) | 103. Busyness |
|  | 104. Coarseness |
|  | 105. Complexity |
|  | 106. Contrast |
|  | 107. Strength |

**Table S3**

**Selected Features by LASSO Regression after 10-fold Cross-validation.**

| Features | Standardized Coefficients | ICC 1 | ICC 2（n=46） |
| --- | --- | --- | --- |
| Intercept | 0.7321 |  |  |
| Skewness | 0.0509 | 0.86 | 0.89 |
| glcm_Idn | -0.0327 | 0.96 | 0.96 |
| glcm_Imc1 | 0.0002 | 0.96 | 0.95 |
| gldm_SmallDependenceLowGrayLevelEmphasis | -0.0173 | 0.86 | 0.78 |
| glrlm_ShortRunEmphasis | 0.0049 | 0.99 | 0.99 |
| shape_Flatness | -0.0110 | 0.88 | 0.90 |
| shape_LeastAxisLength | -0.0084 | 0.98 | 0.98 |
| shape_Sphericity | -0.0212 | 0.95 | 0.96 |
| shape_SurfaceVolumeRatio | 0.0266 | 0.96 | 0.96 |

Note. $\mathrm{Ra}diomics score = 0.7321 + 0.0509 * firstorder\_Skewness -0.0327 *glcm\_Idn +0.0002 * glcm\_Imc1 -0.0173 *gldm\_SmallDependenceLowGrayLevelEmphasis +0.0049 * glrlm\_ShortRunEmphasis -0.0110 * shape\_Flatness -0.0084 * shape\_LeastAxisLength -0.0212* shape\_Sphericity +0.02656 * shape\_SurfaceVolumeRat\mathrm{io}$. ICC 1=intra-observer correlation coefficient. ICC 2=inter-observer correlation coefficient.

**Table S4**

**Models' Hyperparameter Settings.**

| Models | Hyperparameters |
| --- | --- |
| Logistic Regression | L1 regularization; C = 0.05 (= 1/λ); solver = liblinear |
| Decision Tree | max_depth = 4; min_samples_split = 2; min_samples_leaf = 5; max_leaf_nodes = 10; min_impurity_decrease = 0.005; criterion = entropy |
| Random Forest | Mtry=20, ntree=500, criterion = Gini |
| Support Vector Machine (SVM) | kernel = RBF; C = 0.009875; gamma = 0.001638 |
| Extreme Gradient Boosting (XGBoost) | eta = 0.3; gamma = 0.001; max_depth = 2; subsample = 0.7; colsample_bytree = 0.4; num_round = 1000; early_stopping = 200 (AUC); objective = binary: logistic |
| K-Nearest Neighbors | k=5, distance = Euclidean; weights = uniform |
| Multinomial Naive Bayes | α=1 |

**Table S5**

**Performance metrics of the Miller-Payne grading prediction model based on radiomics scores**

| Model | Feature Type | Set | ACC (%) | SEN (%) | SPE (%) | PPV (%) | NPV (%) | F1 Score (%) |
| --- | --- | --- | --- | --- | --- | --- | --- | --- |
| LR | Radiomics score | Training | 83 | 59 | 68 | 83 | 39 | 69 |
|  |  | Test | 83 | 57 | 64 | 83 | 33 | 67 |
| LR | Combined features | Training | 87 | 66 | 74 | 87 | 46 | 75 |
|  |  | Test | 90 | 62 | 80 | 90 | 41 | 73 |
| XGB | Combined features | Training | 92 | 77 | 81 | 92 | 58 | 84 |
|  |  | Test | 86 | 71 | 64 | 86 | 42 | 78 |
| DT | Combined features | Training | 88 | 83 | 64 | 88 | 54 | 86 |
|  |  | Test | 80 | 82 | 44 | 80 | 48 | 81 |
| NB | Combined features | Training | 87 | 66 | 74 | 87 | 46 | 75 |
|  |  | Test | 90 | 62 | 80 | 90 | 41 | 73 |

Note. LR= Logistic Regression. XGB=XGBoost. DT= decision tree. NB=Naive_Bayes. combined features= Radiomics score + HER2 status. ACC= Accuracy. SEN= Sensitivity. SPE=Specificity. PPV=Positive predictive value. NPV=Negative predictive value.

**Table S6**

**The optimal radiomics score cutoff determined by ROC analysis**

| Characteristic | Set | Cutoff | AUC | ACC (%) (%) | SEN (%) | SPE (%) | PPV (%) | NPV (%) |
| --- | --- | --- | --- | --- | --- | --- | --- | --- |
| Radiomics score | all dataset | 0.053 | 0.627 | 0.61 | 0.59 | 0.67 | 0.83 | 0.37 |

**Table S7**

**Clinicopathological characteristics stratified by radiomics score cutoff**

| Characteristics | Radiomics Score | | P Value |
| --- | --- | --- | --- |
|  | Low (n =162) | High (n = 174) |  |
| Age（Mean ± SD） | 49.39 ± 10.16 | 48.16 ± 8.88 | 0.24 |
| HR status (n %) |  |  | 0.748 |
| Negtive | 33 (20) | 32 (18) |  |
| Positive | 129 (80) | 142 (82) |  |
| ER status (n %) |  |  | 0.65 |
| Negtive | 55 (34) | 54 (31) |  |
| Positive | 107 (66) | 120 (69) |  |
| PR status (n %) |  |  | 0.603 |
| Negtive | 46 (28) | 44 (25) |  |
| Positive | 116 (72) | 130 (75) |  |
| HER2 status (n %) |  |  | 0.682 |
| Negtive | 116 (72) | 120 (69) |  |
| Positive | 46 (28) | 54 (31) |  |
| Ki67 index (n %) |  |  | 0.046 |
| <20% | 21 (13) | 38 (22) |  |
| ≥20% | 141 (87) | 136 (78) |  |
| Molecular subtype (n%) |  |  | 0.616 |
| HR+/HER2- | 101 (62) | 103 (59) |  |
| HR+/HER2+ | 28 (17) | 39 (22) |  |
| HR-/HER2+ | 18 (11) | 15 (9) |  |
| HR-/HER2- | 15 (9) | 17 (10) |  |
| Clinical T stage (n %) |  |  | < 0.001 |
| T1-T2 | 81 (50) | 145 (83) |  |
| T3-T4 | 81 (50) | 29 (17) |  |
| Clinical N stage (n %) |  |  | 0.008 |
| N0 | 16 (10) | 29 (17) |  |
| N1 | 93 (57) | 112 (64) |  |
| N2-N3 | 53 (33) | 33 (19) |  |
| Treatment response |  |  | < 0.001 |
| MP grades 1-2 | 60 (37) | 30 (17) |  |
| MP grades 3-5 | 102 (63) | 144 (83) |  |

**Supplemental Figures**


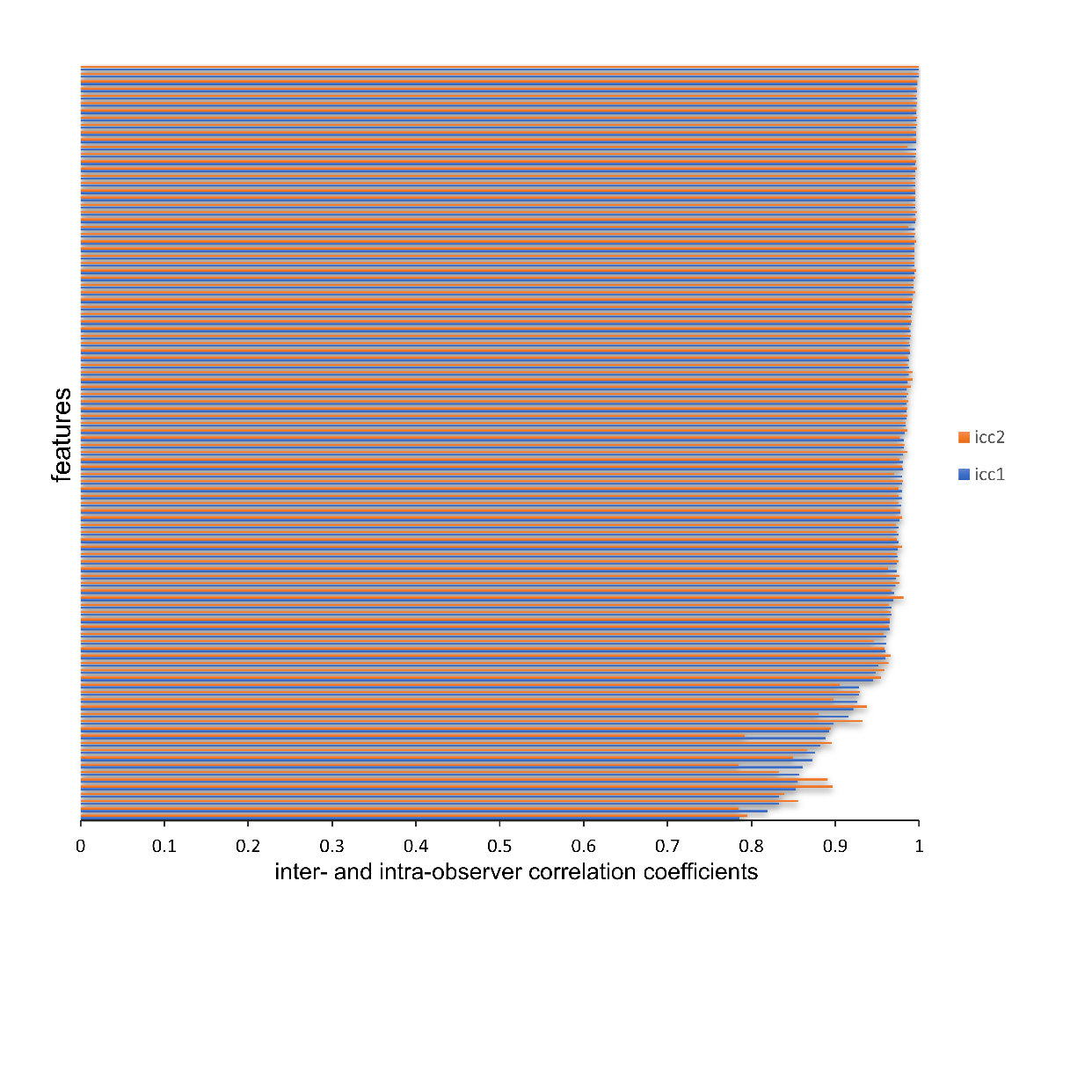


**Fig S1:** inter- and intra-observer correlation coefficients (ICC ≥ 0.75). Each bar represents a radiomics feature. ICC 1=intra-observer correlation coefficient. ICC 2=inter-observer correlation coefficient.


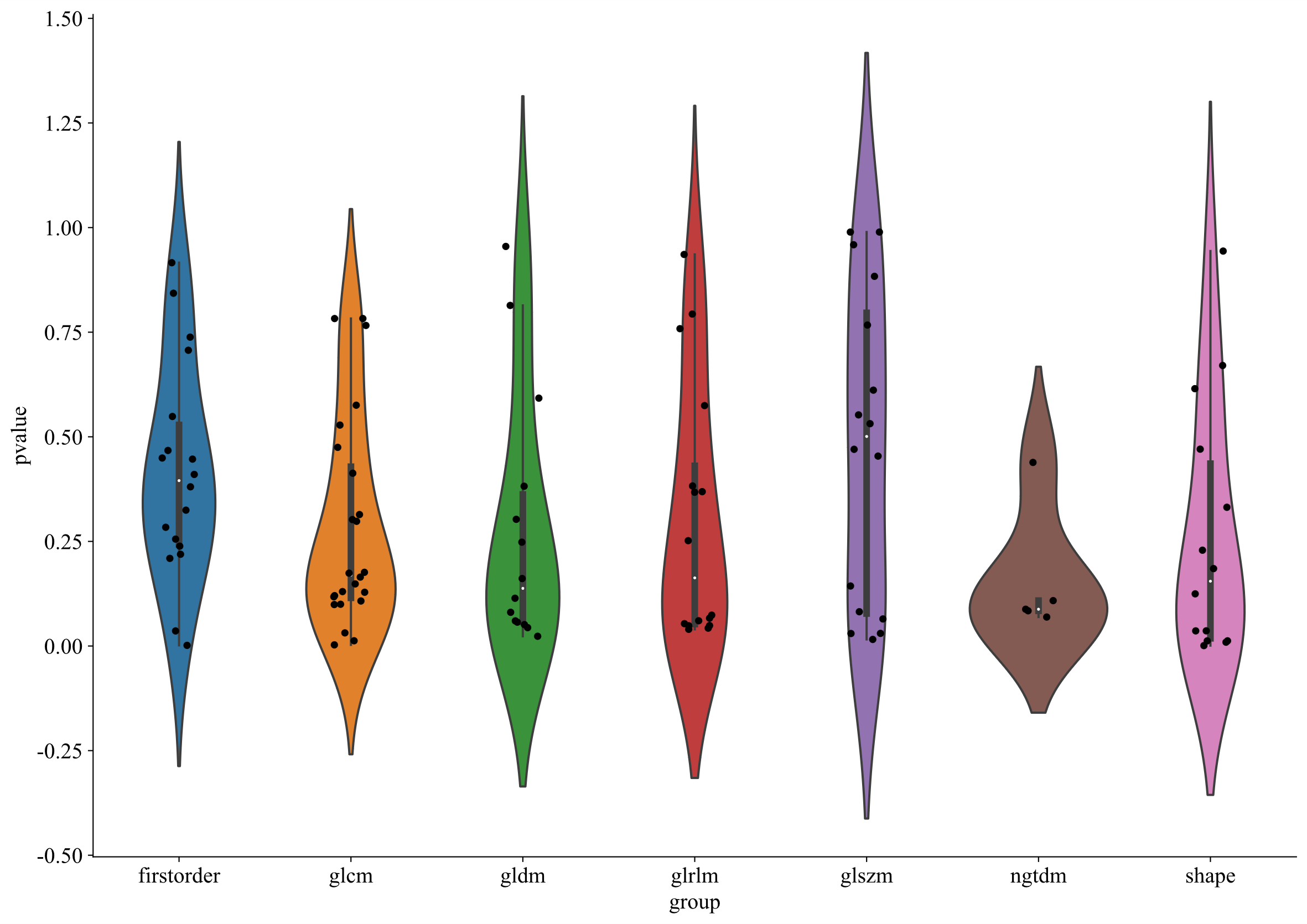


**Fig S2:** Student's t test or Mann-Whitney U test select features associated with MP grading.


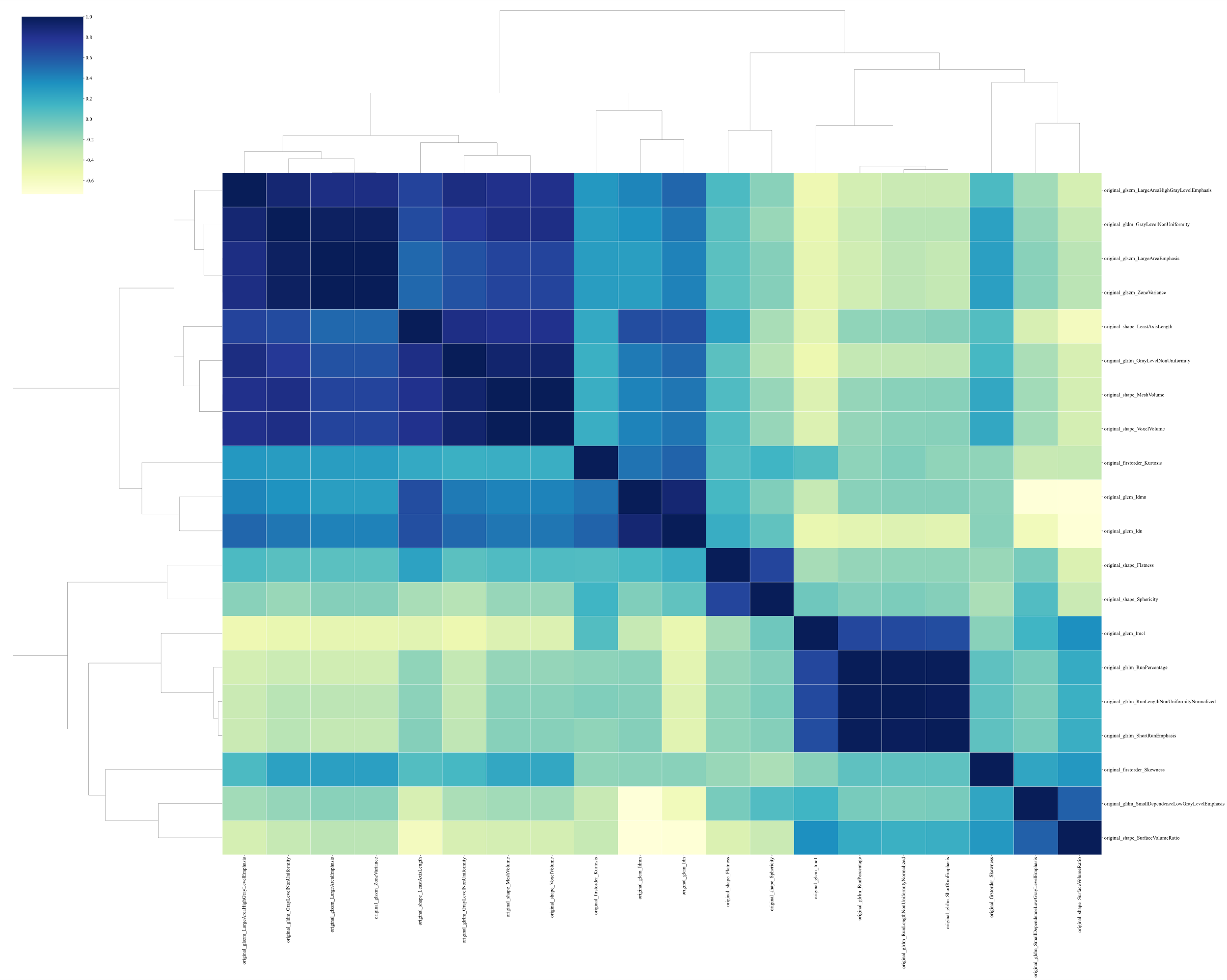


**Fig S3:** Spearman correlation matrix illustrating feature relationships. The color gradient (blue to yellow) represents correlation coefficients ranging from -1 to 1.


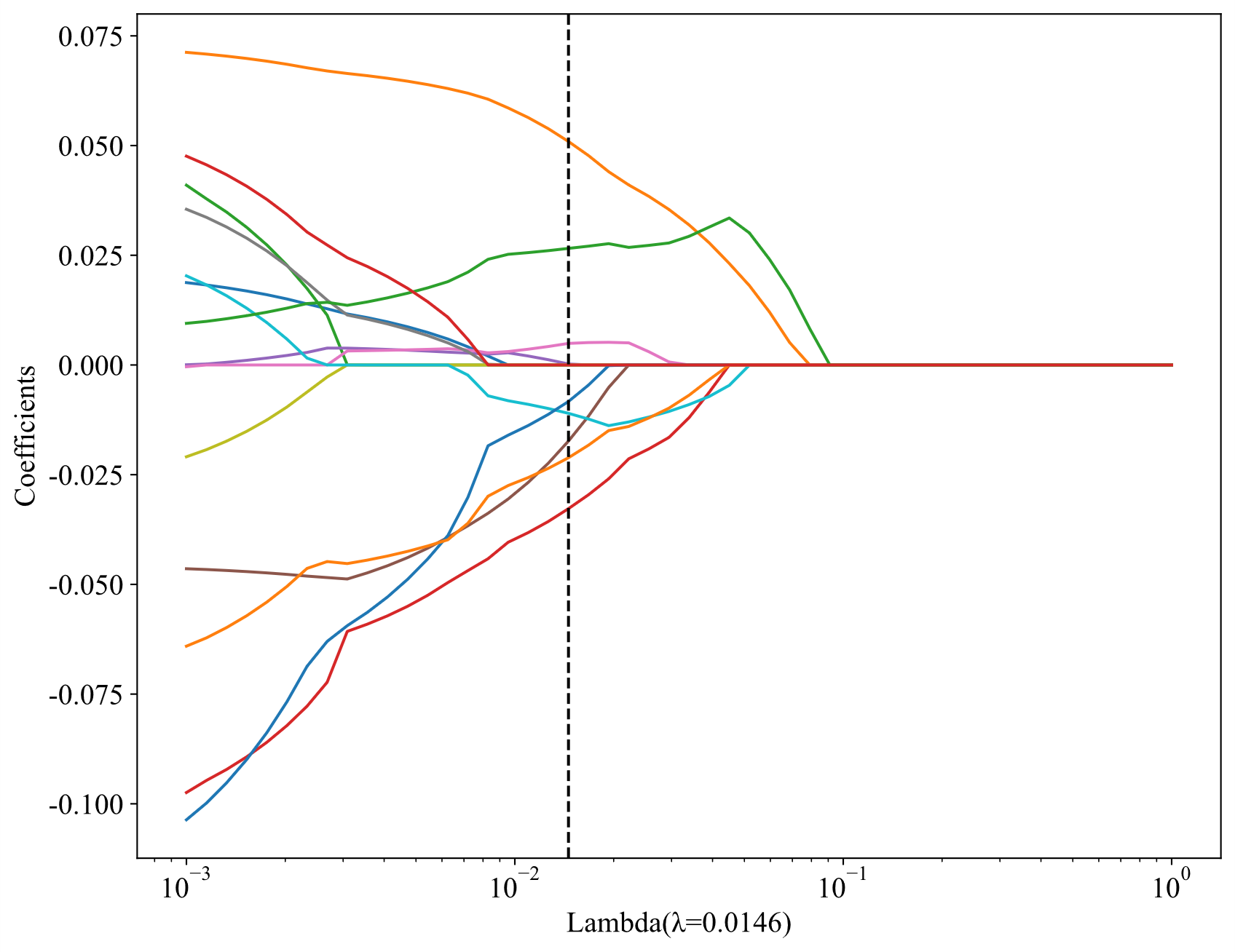


**Fig S4:** the least absolute shrinkage and selection operator (LASSO) algorithm combined with 10-fold cross-validation selected the most discriminative radiomics features.

**Fig S5:** Final selected features and their corresponding weighting coefficients from the LASSO regression model.
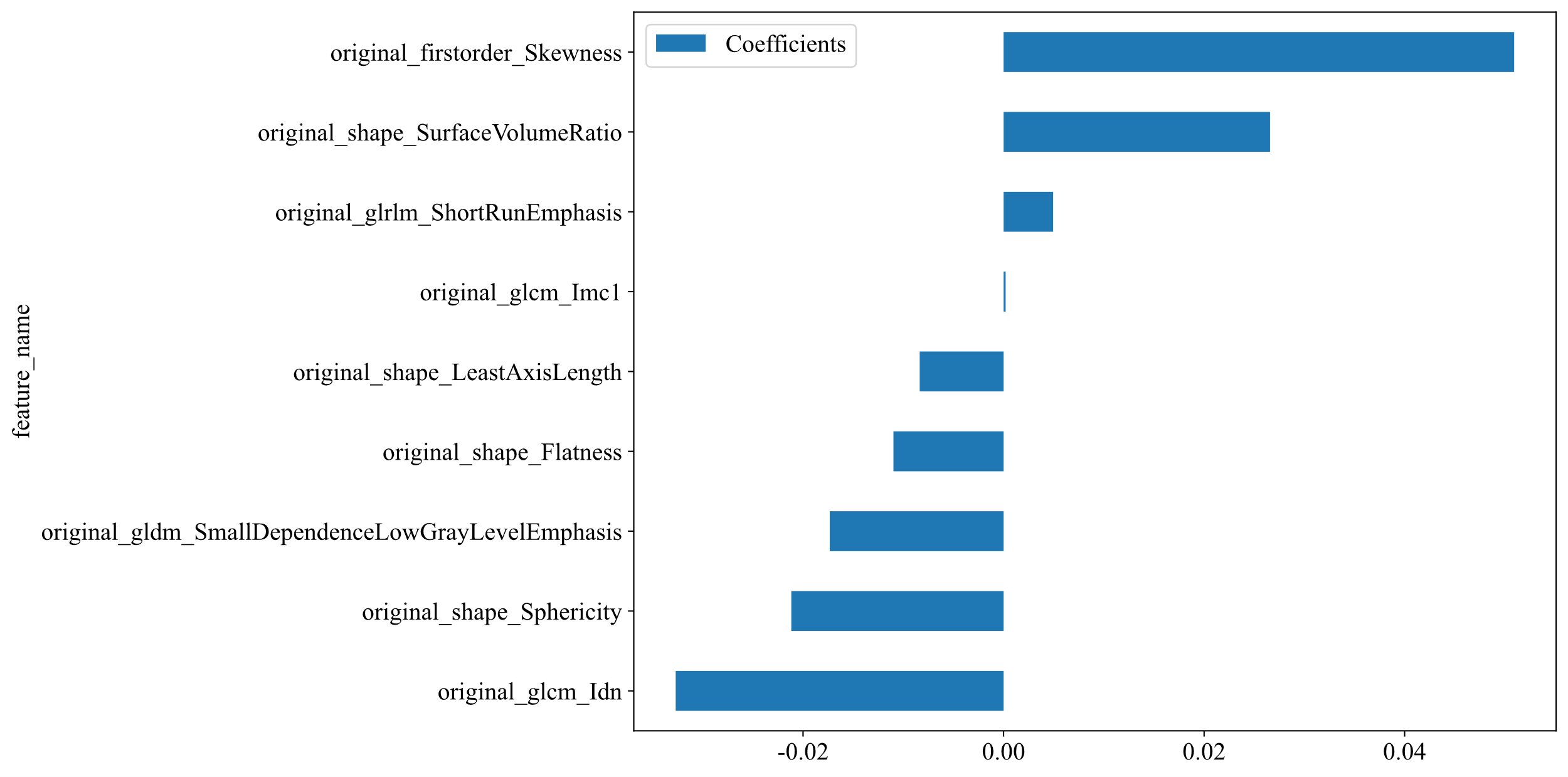

Supplement: Supplementary file 1 — Supplementary Material 1 [file 40644_2026_990_MOESM1_ESM.docx]
